# Current Opinion on Usage of L-Carnitine in End-Stage Renal Disease Patients on Peritoneal Dialysis

**DOI:** 10.3390/molecules24193449

**Published:** 2019-09-23

**Authors:** Mario Bonomini, Lorenzo Di Liberato, Victor Zammit, Arduino Arduini

**Affiliations:** 1 Department of Medicine, Section of Nephrology and Dialysis, G. d’Annunzio University, SS. Annunziata Hospital, 66100 Chieti, Italy; 2 Clinical Sciences Research Institute, Warwick Medical School, University of Warwick, Coventry CV4 7AL, UK; 3 Department of Research and Development, CoreQuest Sagl, Tecnopolo, 6934 Bioggio, Switzerland

**Keywords:** carnitine, end-stage renal disease, peritoneal dialysis, osmolyte

## Abstract

The advantages of peritoneal dialysis (PD) over hemodialysis (HD) are well-documented. Notwithstanding, only a small proportion of patients with end-stage renal disease (ESRD) are managed with PD. This may be related to the high glucose load that PD solutions in current use have on the patient. The effects of such excess glucose include the relatively early limitation of the ultrafiltration capacity of the peritoneal membrane, and the metabolic effects associated with hyperglycemia, e.g., decreased insulin sensitivity. This article describes the advantages that may be realized by the glucose-sparing effects of substituting part of the glucose load with other osmotically active metabolites, particularly L-carnitine. The latter is anticipated to have metabolic advantages of its own, especially as in PD patients, high plasma concentrations can be achieved in the absence of renal clearance. Besides its better biocompatibility, L-carnitine demonstrates anti-anemia action due to its effects on erythropoiesis, and positive effects on the longevity and deformability of erythrocytes. Observations from our trials on the use of carnitine-enriched PD solutions have demonstrated the effectiveness of L-carnitine as an efficient osmolyte in PD, and its favorable effect on the insulin sensitivity of the patients. The significance of these findings for future developments in the use of PD in the management of patients with ESRD is discussed.

## 1. Introduction

Peritoneal dialysis (PD) represents an established home-care, cost-effective modality of renal replacement therapy (RRT) for patients suffering from end-stage renal disease (ESRD). Though prescribed to only a minority of patients requiring dialysis, PD offers several advantages as compared to hemodialysis, including a more gradual and continuous removal of solutes and fluid, minimal cardiac stress, a better preservation of residual renal function, and similar survival [1]. When considering that ESRD requiring RRT is a growing problem worldwide and that it represents a significant economic burden to any health system [2], it is likely that PD (which is less expensive than hemodialysis) will become more and more frequently used over the next years.

PD uses the semipermeable peritoneum as a biological dialysis membrane [3]. Removal of fluid and exchange of solutes between the peritoneal capillaries and a solution introduced into the peritoneal cavity via an implanted intra-abdominal catheter is the blood depuration mode (dialytic exchange) PD is based on. After a dwell time of 4 to 8 h, the solution is drained out, and fresh dialysate is re-infused. This can be performed manually (continuous ambulatory PD; CAPD) with on average four exchanges daily, or by employing a cycler (automated PD; APD) during the night (on average 8 h). Removal of excess water and retained uremic solutes provides benefits to the ESRD patient. However, removal is nonspecific, which may lead to unwanted depletion of useful substances.

PD solution (also called PD fluid; dialysate) is composed of physiological concentrations of electrolytes (sodium, calcium, magnesium, chloride), a buffer (lactate and/or bicarbonate), and an osmotic agent needed to remove excess water from the patient (peritoneal ultrafiltration). Fluid can be drawn from the peritoneal capillary blood through both the water-exclusive aquaporins and the small-pore, solute-coupled fluid pathways [4]. Failure of ultrafiltration capacity is the most frequent abnormality in long-term PD, and the main reason of technique failure [5].

Glucose is the standard osmotic agent used in PD solution. It has a molecular weight of 180 Da, a high osmotic capacity, low cost, an acceptable safety profile, and represents an energy source for patients who are often not well nourished. However, prolonged exposure to the high glucose content in PD solution is thought to be associated with acceleration of the progressive changes in peritoneal membrane function, causing ultrafiltration failure [6]. Furthermore, excessive intraperitoneal glucose absorption has the potential to cause many systemic metabolic side effects in PD patients, including insulin resistance, new onset diabetes, and cardiovascular disease [7,8,9]. The development of glucose-sparing strategies able to provide efficacious peritoneal ultrafiltration without jeopardizing patients’ health represents a major challenge in present-day PD therapy [10].

L-carnitine has become of interest in ESRD because decreased renal synthesis and dialysis losses have been suggested as causes of L-carnitine deficiency in patients on hemodialysis [11]. In turn, dialysis-related carnitine deficiency has been associated with several symptoms frequently encountered in uremic patients including anemia, cardiomyopathy, skeletal muscle weakness, and fatigability [11,12]. Though routine carnitine supplementation in uremic patients is not recommended [12,13], symptomatic dialysis patients who do not respond to standard therapy may benefit from carnitine supplementation [12,14].

There have been relatively few reports on carnitine in ESRD patients receiving PD therapy. In this article, we have reviewed the available evidence for the status of L-carnitine and its potential therapeutic use in PD.

## 2. L-Carnitine in Peritoneal Dialysis

### 2.1. Metabolism

L-carnitine is a highly water soluble compound (>2500 g/L at 20 °C) which, owing to the presence of both a positively charged quaternary ammonium group and a carboxylate group, exists as zwitterion at pKa of 3.8 when dissolved in water at 20 °C.

Carnitine metabolism may be significantly altered in ESRD patients on PD as compared to healthy controls. Abnormal plasma levels for free and acetyl-carnitine have been reported in several studies in both adult [15,16,17] and pediatric [18,19] patients on CAPD, and in reports on patients treated with APD [17,20]. In addition, we also found significant differences between healthy subjects and PD patients for other carnitine species, showing a clear increase in a subset of short-chain dicarboxylic acylcarnitine esters [17]. Other reports, however, contain conflicting results as to blood levels of the main carnitine species in CAPD patients [21,22,23,24]. These discrepant results may be at least partially explained by differences in the carnitine measurement method, study design, dietary habits, patient population, and lack of control data [17].

Carnitine metabolism disorders in ESRD patients on PD may be attributed to several mechanisms, including inadequate dietary intake, reduced renal synthesis, accumulation of metabolism intermediates, intestinal malabsorption, and dialytic age [11,17,25,26]. Daily losses in dialysate during dialysis dwell time may also contribute to carnitine disturbances [16,17,20,24]. Indeed, unlike the natural kidney, the peritoneal membrane functioning as a filter lacks both the selectivity for preferential retention of free L-carnitine to acyl-L-carnitine and the ability to conserve L-carnitine when plasma levels decrease, thereby disrupting carnitine homeostasis [25]. Peritoneal membrane function and dialysis technique may also play a role in carnitine metabolism of PD patients. A better carnitine metabolic status has in fact been observed in patients with high as compared to low peritoneal transport rates [27]. In addition, lower levels of free and acetyl-carnitine in patients treated with APD as compared to CAPD have been found [17]. The different nature of the PD depurative treatments may lie behind this finding, since APD is characterized by shorter and larger volume dwells, which might favor the removal of carnitine species [17].

### 2.2. Studies on Oral Supplementation of L-Carnitine

Taking into account the vital role of L-carnitine in fatty acid metabolism, and given the derangement of the carnitine system in ESRD patients on PD, several studies have tested the effects of oral carnitine supplementation (Table 1).

Some observational studies have suggested a possible association in PD patients between abnormalities of the carnitine system and of lipid metabolism [15,23,27]. In the sole randomized trial to date, Waradi et al. investigated the effects of oral L-carnitine (100 mg/kg/day) administered for 2 months to six pediatric patients receiving CAPD [22]. The mean plasma carnitine concentration significantly increased, but no change was found in serum triglyceride levels [22]. Kosan et al. evaluated the effects of L-carnitine (50 mg/kg/day) given orally for 30 days to 20 children on PD [18]. At the end of the study, a significant decrease in serum apolipoprotein B levels was observed, with no change in the other lipid parameters measured. Because elevated apolipoprotein B levels may represent an additional risk factor for the development of atherosclerosis and coronary artery disease [30,31,32], the authors suggested considering carnitine supplementation in children on PD to antagonize cardiovascular complications [18]. These results, however, were not confirmed in a subsequent study in 13 children on APD receiving 20 mg/kg/daily L-carnitine for 3 months [20]; carnitine supplementation restored free carnitine levels and produced a positive carnitine balance without significant effects on lipid profile [20].

Other studies have assessed the effects of carnitine supplementation on hematological parameters relative to anemia and on the requirement of human recombinant erythropoietin (rHuEPO). Normochromic normocytic anemia is a frequent and serious complication in uremic patients, which reduces the quality of life and is associated with many adverse clinical consequences, and is primarily treated with erythropoiesis-stimulating agents such as rHuEPO and adjuvant iron therapy [33]. The anti-anemic action of L-carnitine is based on the biophysical, metabolic, and anti-apoptotic effects of this compound on erythropoiesis and the function of circulating erythrocytes [34].

Sotirakopoulos et al. examined the effect of L-carnitine (2 g daily per os) on hematocrit and hemoglobin levels in 12 adult patients on CAPD [29]. A significant increase of hematocrit (38.1 ± 3.4 % vs. 35.4 ± 3.3% at the beginning of the study, *p* < 0.03) and hemoglobin levels (11.9 ± 1 mg/dL vs. 11 ± 1.1 mg/dL, *p* < 0.01) was found, paralleled by a significant reduction in rHuEPO dose necessity/patient/week (from 3833 ± 3326 to 1292 ± 1712, *p* < 0.01). Interestingly, the rigidity index of erythrocytes proved to be significantly decreased, suggesting an improvement of their deformability [29]. Deformability of erythrocytes, which is impaired in chronic uremia [35], is fundamental for their survival in the blood stream and can influence tissue oxygenation. Amelioration of erythrocyte deformability by L-carnitine administration, as previously reported in hemodialysis patients [36], can improve red cell survival and hence increase hematocrit values [36]. Positive results on hematological parameters and rHuEPO requirement following oral L-carnitine administration were, however, not confirmed in the study of Verrina et al. in a pediatric APD population [20].

It is difficult to combine the results so far obtained (Table 1) in order to reach any definite conclusions regarding the oral use of L-carnitine in PD patients, given the small number of patients enrolled, lack of control data, variability of doses, and duration of supplementation. The oral route of administration might also have influenced the apparently small effect sizes of L-carnitine therapy in PD. Indeed, the pharmacokinetic properties of L-carnitine, as compared to a traditionally orally administered drug, are relatively unattractive and complex: very poor intestinal absorption, high renal clearance, and tissue uptake via a high affinity transporter [25,37,38]. Even when a high oral L-carnitine dosage is administered, only a very modest increase of L-carnitine plasma and target organ exposures occurs [37]. In addition, physiological plasma levels of L-carnitine will tend to saturate the high affinity L-carnitine transporters, which makes it difficult to expand the intracellular L-carnitine pool in target organs (skeletal muscle, heart, liver) where it is in the high micromolar to low millimolar range, whereas in the plasma it is in the low micromolar range [39]. This translates into the need for relatively high L-carnitine plasma exposure to raise L-carnitine intracellular exposure remarkably [39].

Another limitation of the studies done to date on oral L-carnitine supplementation in PD patients has been the short follow-up period, hampering the assessment of hard outcomes including mortality and cardiovascular disease. At the present time, the available data do not allow high expectations from oral L-carnitine supplementation in patients treated with PD.

Oral L-carnitine administration to PD patients is able to increase and normalize free carnitine levels [18,20], in spite of an ongoing loss of the carnitine ester into the dialysate [20]. Use of L-carnitine in higher dosages [18,22] is not associated with higher plasma free carnitine levels than when using a lower dosage [20]. Within this context, it should be emphasized that attempts to restore L-carnitine levels to a normal reference interval as calculated in non-uremic healthy populations may be misguiding, since such “normal” concentrations may be not adequate for the particular metabolic needs of ESRD patients on dialysis [34]. For example, in PD patients with “normal” B12 or folate levels accompanied by elevated homocysteine levels, additional supplementation of B12 or folate may significantly decrease homocysteine, which suggests the need for concentrations above the normal reference intervals of these vitamins [40]. A large body of experimental and clinical data indicates that L-carnitine at concentrations higher (low millimolar range) than those physiologically present in the extra- and intra-cellular milieu (low micromolar to low millimolar range) exerts favorable pharmacological actions in vitro and in vivo [39,41]. Very high plasma L-carnitine exposure has been safely achieved in HD [42] and PD [43] ESRD patients treated with L-carnitine for a prolonged time period. Supra-physiological concentrations of L-carnitine in plasma and target organs may exert beneficial effects on several parameters that have derangements of a common origin (e.g., type 2 diabetes, insulin resistance, dyslipidemia) and which are frequently present in ESRD patients on dialysis [39]. As a matter of fact and as detailed below, a significant improvement of insulin sensitivity was observed in patients on CAPD treated for 4 months with an L-carnitine-enriched PD fluid [43].

### 2.3. Studies on L-Carnitine Addition to PD Fluid

The poor local (peritoneum cavity) and systemic biocompatibility of the currently available glucose-based solutions for PD is increasingly being recognized [5]. Since glucose is thought to be the main culprit behind the bioincompatibility of PD solution [44], several alternative osmotic agents have been examined over the years, but only two agents are currently available in glucose-free solutions for PD clinical practice: the glucose polymer icodextrin and amino acids. These formulations, either alone or in combination, have proven to be effective and their use may benefit PD patients [44,45,46,47,48]. However, both icodextrin and amino acids can only replace 30–50% of the daily glucose absorption [44], and their use is limited to a single daily peritoneal exchange [49,50]. Moreover, two recent randomized trials in diabetic PD patients showed that combined use of icodextrin and amino acids, though it improved metabolic indices, was associated with an enhanced risk of extracellular fluid volume expansion, causing an increase in serious adverse events and deaths [47]. Limited progress has been achieved to date in PD fluid technology [5]. However, the need is clear for new solutions able to minimize the negative effects of PD.

L-carnitine has a molecular weight of 161.2 Da and is highly water soluble and chemically stable in aqueous solutions [51], which render it suitable for use in PD fluid.

When developing a new PD solution for clinical use, one should first examine its impact on the peritoneal membrane, which consists of three layers: the mesothelium, the interstitium, and the capillary endothelium [52]. Several investigations have shown PD solution containing carnitine to be more biocompatible than standard glucose-based solutions. In vitro and in vivo (rabbit model), PD solution containing carnitine was associated with better growth and less cytotoxicity to mesothelial cells [53]. Addition of L-carnitine to glucose-based PD solutions significantly improved murine fibroblast L929 viability [54], a standard toxicity test [55]. In addition, in cultured human umbilical vein endothelial cells, the addition of L-carnitine prevented glucose-induced apoptosis, did not affect the percentage of early apoptotic cells, and significantly improved cell viability [54].

Clinical studies examining the effects of L-carnitine added to PD fluid are reported in Table 2.

Bazzato et al. examined the effects of 2 g L-carnitine added to the PD fluid for the nocturnal exchange in seven CAPD patients [56]. Plasma levels of carnitine were restored, and the lipid pattern was ameliorated in six patients [56]. In five CAPD patients receiving 20 mg/kg L-carnitine in their first daily PD solution for 14 days, an improvement in nitrogen balance was observed [57].

Since L-carnitine has been shown to possess osmotic properties in different biological systems [58,59], we evaluated the osmotic properties of L-carnitine addition to PD fluid [54]. In rat and mouse models, equiosmolar solutions of L-carnitine and glucose induced a comparable amount of net peritoneal ultrafiltration. Subsequently, four stable patients on CAPD received for five consecutive days a PD solution for the nocturnal exchange containing L-carnitine (5 g, 0.25%) and glucose (1.5%), replacing a 2.5% glucose-based solution routinely used by patients. Use of the experimental solution was associated with higher net peritoneal nocturnal ultrafiltration than that achieved with the 2.5% glucose solution, despite the lower osmolarity of the carnitine-containing solution [54].

Our results indicate that L-carnitine has potential use as a new osmotic agent in PD solution [54]. The capacity shown by L-carnitine for removing fluid from the peritoneal cavity might be related to its physicochemical properties. L-carnitine has a close molecular weight to glucose and is a zwitterionic molecule, a feature that may affect osmotic processes across the peritoneal membrane. Alternatively, but not mutually exclusively, L-carnitine ultrafiltration capacity might also be related to a favorable action on the water channel aquaporin-1. According to the three-pore model, which describes the function of the peritoneal membrane well, the major transport barrier of the membrane is the capillary endothelium, which contains small, large, and ultrasmall pores [60], the latter being the molecular counterpart of aquaporin-1 [61]. Studies in a mouse knockout model for aquaporin-1 showed that, as for glucose, about half of the ultrafiltration generated by L-carnitine contained in the PD fluid reflected facilitated water transport by aquaporin-1 [54]. Importantly, the addition of L-carnitine to endothelial cells in culture significantly increased the expression of aquaporin-1, and significantly reverted the inhibitory effect of glucose on aquaporin-1 protein levels [54].

Several studies have shown the beneficial effects of L-carnitine administration in mitigating insulin resistance or even improving glycaemic control in diabetic animal models or in humans [62]. It has also been suggested that oral L-carnitine administration may accelerate atherosclerosis via gut microbiota metabolites, although this is still very controversial [63,64]. In addition, a systematic review and meta-analysis of several controlled trials showed that the oral administration of L-carnitine led to a 27% reduction in all-cause mortality, a 65% reduction in ventricular arrhythmias, and a 40% reduction in angina symptoms in patients experiencing an acute MI as compared to placebo [65]. In HD patients, a single i.v. administration of L-carnitine improved insulin sensitivity, as evaluated by an insulin tolerance test [66]. Moreover, in a randomized, matched-paired, double-blind, placebo-controlled experimental design, the effect of chronic intravenous L-carnitine supplementation in HD patients in modifying insulin resistance and protein catabolism was investigated [67]. L-carnitine treatment resulted in a statistically significant reduction of leucine oxidation rates and appearance from proteolysis during the clamp studies compared to the placebo group. At variance, insulin-mediated glucose disposal was significantly improved by L-carnitine only in those patients with greater baseline insulin resistance, selected according to the median value of insulin sensitivity before treatment.

Recently, we evaluated in a multicenter, randomized controlled trial the effects of an L-carnitine-containing PD solution on insulin sensitivity (as determined by performing a euglycemic hyperinsulinemic clamp) in CAPD patients [43]. Insulin resistance is frequently present in ESRD patients and may cause enhanced morbidity and mortality through an increased occurrence of cardiovascular disease [8,68]. Nondiabetic CAPD patients were randomized to receive diurnal PD exchanges with either a standard glucose-based solution or a solution with an identical glucose amount enriched with L-carnitine (2 g per each daily bag) for 4 months. The presence of L-carnitine in PD fluid was well tolerated by patients (*n* = 15), and was associated with a significant improvement in insulin sensitivity, as compared to both baseline and results in the control group (*n* = 12). Interestingly, while in the control group daily urine output was significantly decreased, diuresis did not change in the intervention group, a finding which may be explained by L-carnitine-increased urinary excretion (osmotically driven maintenance of urine volume), and may be of clinical relevance, since urine output is quite important in maintaining fluid balance in PD patients [69]. Plasma levels of L-carnitine markedly increased during L-carnitine treatment, achieving an apparent steady state after 30 days, which suggests an apparent equilibrium between L-carnitine absorption, exposure, and excretion in both urine and drained PD fluid. The insulin-sensitizing effect of the L-carnitine-containing solution might be related to the supra-physiological plasma levels of carnitine achieved in the study [43].

As APD has become increasingly popular as a PD modality, we next explored the feasibility of adding L-carnitine to the PD solution in APD-treated ESRD patients [17]. The peritoneal membrane of patients on APD is more intensively and frequently exposed to fresh dialysate, since they receive larger volumes and more frequent, but shorter, dialysis exchanges than patients on CAPD. Five patients on APD receiving three 1.5% glucose bags for their night exchanges in the tidal modality and icodextrin during the daytime, were supplemented with L-carnitine (5 g) in the first solution bag for five consecutive days. L-carnitine addition to PD fluid was associated with stability in several laboratory, physical, metabolic, and dialysis efficiency parameters [17]. Appropriate concentrations of L-carnitine in the dialysate mix components and/or its administration in more than one solution bag remain to be defined in further studies.

Actually, there are no data on hard outcomes for L-carnitine-containing PD solutions. These solutions are, however, still under investigation.

In all studies performed so far (Table 2), L-carnitine-containing solution has proven to be safe and well tolerated, and adverse events have not been attributable to treatment. The only potential side-effect from L-carnitine administration may derive from a significant increase of plasma osmolarity, as observed with glycerol [70], though this condition may only be achieved by using huge doses of L-carnitine in the PD solution.

L-carnitine in PD fluid may be envisaged as a prototypical osmo-metabolic agent. Osmo-metabolites can be defined as those substances exhibiting both osmotic and metabolic favorable properties [71,72]. The novel osmo-metabolic approach to the composition of PD solutions would ensure not only a reduction of the intraperitoneal glucose load without compromising ultrafiltration, but also the independent mitigation of underlying metabolic disorders—a sort of bioactive glucose sparing.

Osmo-metabolic agents may also be used in combination, in order to maximize their therapeutic effects. We have recently developed a new PD solution containing L-carnitine (1.24 mmol/L), xylitol (46 or 98.6 mmol/L), and a low amount of glucose (27.7 mmol/L) [72]. Xylitol, another osmo-metabolite, is a five-carbon sugar alcohol (pentitol) produced by the reduction of D-xylulose. It is involved in the pentose phosphate shunt and has low glycaemic properties [73]. For example, the glycaemic index of xylitol is much lower than that of glucose, a property that makes it more favorable if the caloric load needs to be better controlled, as in PD patients. Xylitol entry into cells is insulin-independent, it is efficiently metabolized after being intravenously infused with no elevation of glycaemia, it stimulates much less insulin secretion than does glucose [74], and it is less of an irritant to the veins than is glucose when given in hyperosmolar solution [75]. Since 1970, xylitol has been used in diabetics and for parenteral nutrition as a glucose substitute in critically ill patients (i.e., post-traumatic, septic patients), though in the majority of cases it has been administered in combination with other sugars such as glucose and fructose [76]. In a clinical trial many years ago [77], six insulin-dependent diabetic patients on CAPD were switched from a glucose-based PD regimen to a daily therapeutic program with D-xylitol as the sole osmotic agent (three daily exchanges of PD solution with xylitol 1.5% and one exchange with xylitol 3%). After a follow-up of at least five months, use of xylitol-containing PD fluid proved to be safe, maintained peritoneal ultrafiltration, and significantly improved the patients’ glycaemic control (half-dosage of exogenous insulin requirement, significant decrease of glycosilated haemoglobin).

In human endothelial cell models, such an experimental PD solution [72] did not cause the cytotoxicity, nitro-oxidative stress, and inflammation caused by a glucose-based, neutral pH, low-glucose degradation product PD solution, which is regarded as a “biocompatible” solution [78]. Two clinical trials with new PD solutions based on L-carnitine, xylitol, and low glucose are under advanced development (ClinicalTrials.gov Identifier: NCT04001036 and NCT03994471).

## 3. Conclusions

ESRD patients on PD treatment frequently suffer from abnormalities of the carnitine system, which might contribute to the complex pathophysiology of uremic syndrome. Oral supplementation of L-carnitine does not appear to be able to ensure the plasma carnitine exposure needed to trigger a beneficial effect in target organs. However, this may be achieved through the safe use of L-carnitine added to the PD fluid. Patients suffering from ESRD on PD therapy may benefit from a glucose-sparing approach, in terms of solution prescription [79]. The use of L-carnitine as an osmo-metabolic agent in PD solution represents a novel and tantalizing tool to antagonize glucose-associated toxicity. This year, L-carnitine-containing PD fluid received a very favorable opinion for marketing authorization by the German Federal Institute for Drugs and Medical Devices (BfArM, Germany).

The clinical practice will ultimately clarify and define the appropriate use of osmo-metabolic enriched PD solutions for PD therapy.

## Figures and Tables

**Table 1:** Summary of studies on oral L-carnitine treatment in end-stage renal disease patients on peritoneal dialysis.

Study	Study Design	Patient Population	L-Carnitine Treatment	Duration of Treatment	Results
Warady et al. [22]	Randomized, no placebo-controlled	6 CAPD (pediatric)	100 mg/kg/day	2 months	No change in triglycerides
Kosan et al. [18]	Open-label, no control group	20 CAPD (pediatric)	50 mg/kg/day, two divided doses	30 days	Decreased apolipoprotein B
Verrina et al. [20]	Open-label, no control group	13 CAPD (pediatric)	20 mg/kg/day, two divided doses	3 months	Positive carnitine balance, no effects on hematological or lipid parameters
Lilien et al. [28]	Open-label, no control group	4 CAPD (pediatric)	20 mg/kg/day, two divided doses	26 weeks	No change in rHuEPO ^1^ requirement
Sotirakopoulos et al. [29]	Open-label, no control group	12 CAPD (adult)	2 g/day	3 months	Increased hematocrit and hemoglobin levels; decreased rHuEPO dose

^1^ rHuEPO = human recombinant erythropoietin.

**Table 2:** Summary of studies on L-carnitine-containing solution in end-stage renal disease patients on peritoneal dialysis.

Study	Study Design	Patient Population	L-Carnitine Treatment	Duration of Treatment	Results
Bazzato et al. [56]	Open-label, no control group	6 CAPD	2 g/day in the PD solution for nocturnal exchange	2 months	Improved lipid pattern in 6 patients
Kopple and Qing [57]	Open-label, no control group	5 CAPD	20 mg/kg/day, in the first daily PD solution	14 days	Improved nitrogen balance
Bonomini et al. [54]	Open-label, no control group	4 CAPD	5 g in the PD solution for nocturnal exchange	5 days	Increased net peritoneal nocturnal ultrafiltration
Bonomini et al. [43]	Randomized, single-blind, control group	27 CAPD (standard solution, *n* = 12; experimental solution, *n* = 15)	2 g in the PD solution for diurnal exchanges	4 months	Increased insulin sensitivity, maintenance of urine output
Di Liberato et al. [17]	Open-label, no control group	5 APD	5 g in the first solution bag of night exchanges	5 days	Stable laboratory, metabolic and dialytic parameters
